# Constructing the optimal experimental autoimmune thyroiditis mouse model using porcine thyroglobulin

**DOI:** 10.3389/fimmu.2025.1591196

**Published:** 2025-08-20

**Authors:** Ke Liu, Pei Zhang, Zi-Shan Jin, Xiang-Kun Meng, Jin-Li Luo, Lin Han, Xiao-Tong Yu

**Affiliations:** ^1^ Guang’anmen Hospital, China Academy of Chinese Medical Sciences, Beijing, China; ^2^ South District of Guang’anmen Hospital, China Academy of Chinese Medical Sciences, Beijing, China; ^3^ Beijing University of Chinese Medicine, Beijing, China; ^4^ Changchun University of Traditional Chinese Medicine, Changchun, Jilin, China; ^5^ China Traditional Chinese Medicine Holdings Co Limited, Foshan, Guangdong, China; ^6^ Guangdong e-fong Pharmaceutical CO., LTD., Foshan, Guangdong, China

**Keywords:** autoimmune thyroiditis, experimental autoimmune thyroiditis, NOD/LtJ mice, animal model construction, molecular mechanism

## Abstract

**Introduction:**

Autoimmune thyroiditis (AIT) is a chronic autoimmune disease characterized by lymphocytic infiltration of the thyroid gland and elevated specific antibodies. Its incidence rises annually, yet no standardized animal model fully mimics human AIT. Given unclear pathogenesis and lack of targeted immunotherapies, researchers invest significant time in developing suitable models. This study systematically compares pathological and immunological effects of different immunization conditions (antigen dose, frequency, administration methods) in NOD/LtJ mice to establish an optimal model for elucidating AIT pathogenesis and therapies.

**Methods:**

Eighty female NOD/LtJ mice were divided into subcutaneous (SC) and tail vein intravenous (IV) injection groups. SC groups received porcine thyroglobulin (pTg) emulsified in CFA (primary) and IFA (booster), with doses of 50/100/200 μg and frequencies of 2 or 3 immunizations. IV groups received pTg in PBS followed by LPS (3 immunizations: weeks 1, 3, 4). All model groups drank 0.05% NaI water. Thyroid histopathology (HE staining, infiltration scoring), serum TPO-Ab/TG-Ab (ELISA), cytokines (multiplex assay), Th17/Treg cells (multiplex immunofluorescence), and thyroid IL-17A/NLRP3/Caspase-1 (immunohistochemistry) were analyzed 2 weeks post-last immunization.

**Results:**

High-dose antigen (200 μg pTg) with high-frequency immunization (three times) via SC or IV routes induced severe thyroid lymphocyte infiltration (scores: SC 3.4±0.55, IV 3.2±0.45; p<0.01 vs. controls), follicular destruction, and elevated serum antibodies (TPO-Ab: IV 438.8±13.15 > SC 406.2±7.46; TG-Ab: IV 158.4±5.32 > SC 141.9±2.36). This protocol activated Th1/Th17 cytokines (IL-17A, IL-6, TNF-α), increased Treg cells (p<0.001), and specifically enhanced NLRP3 (p<0.001) and Caspase-1 in thyroid tissue, with IV injection showing superior antibody production and inflammasome activation.

**Discussion:**

The combination of high-dose pTg (200 μg) and three immunizations maximally induced AIT pathology and immune responses in NOD/LtJ mice. Tail vein injection excelled in stimulating antibody production and NLRP3 activation, while subcutaneous injection promoted stronger histological inflammation. After balancing operational feasibility, pathological reproducibility, and immunological specificity, three subcutaneous and intravenous tail injection of 200 μg pTg are recommended as the optimal modeling protocol. This approach accelerates model selection, improves experimental efficiency, and reduces animal use, providing a robust foundation for AIT research.

## 1 Introduction

Autoimmune thyroiditis (AIT), primarily encompassing Hashimoto’s thyroiditis (HT), is an organ-specific autoimmune thyroid disease characterized by thyroidal lymphocyte infiltration and elevated thyroid peroxidase antibody (TPO-Ab) or thyroglobulin antibody (TG-Ab) levels (1). Its pathogenesis involves complex interactions between genetic susceptibility, environmental and epigenetic factors that dysregulate immunoinflammatory responses (2–4). Epidemiological investigations indicate a global annual increase in AIT incidence, with females exhibiting fourfold higher disease susceptibility than males (5). A Chinese cross-sectional study revealed an AIT prevalence rate of 14.19%, significantly surpassing that of Graves’ disease and establishing AIT as the most prevalent autoimmune thyroid disease (6). Clinically, AIT progression leads to thyroid follicular cell destruction, hypothyroidism, and frequent comorbidity with other autoimmune conditions, necessitating deeper mechanistic exploration (7, 8). Current therapeutic strategies predominantly rely on levothyroxine replacement therapy, while the lack of etiological treatments targeting immunoinflammatory pathways underscores the urgency for preclinical pharmacological validation of candidate drugs (9). This context establishes the development of reliable AIT animal models as a pressing research priority (10).

The animal models of AIT currently primarily include experimental autoimmune thyroiditis (EAT) (11) and spontaneous autoimmune thyroiditis (SAT) (12). EAT is typically established by immunizing susceptible mice with thyroglobulin (Tg) combined with lipopolysaccharide (LPS) or complete Freund’s adjuvant (CFA), while SAT is commonly observed in NOD.H-2^h4^ mice (13). This mouse strain is derived from a cross between non-obese diabetic (NOD) mice and B10.A(4R) mice, expressing the H-2K^k^ and I-A^k^ genes on an NOD genetic background. These mice do not develop diabetes but spontaneously develop autoimmune thyroiditis (14). NOD.H-2^h4^ mice are sourced exclusively from the Jackson Laboratory in the United States, and due to the uniqueness of this strain, they are expensive and have a long shipping time. In contrast, NOD/LtJ mice, which share a similar genetic background and are more readily accessible, serve as an ideal alternative to NOD.H-2^h4^ mice and have been utilized in multiple laboratories for research on autoimmune-related diseases (15–17). NOD/LtJ mice were initially bred for studies on autoimmune type 1 diabetes (18, 19) and exhibit high susceptibility to multi-organ autoimmune responses due to their unique genetic background, including the major histocompatibility complex (MHC) haplotype H2-Ag^7^ (20). This susceptibility manifests as autoimmune damage in organs such as the thyroid, salivary glands, and ovaries (21–23). The thyroiditis phenotype in these mice can be induced by immunization with thyroid antigens, characterized by lymphocyte infiltration in the thyroid, destruction of follicular structures, and elevated serum levels of thyroid-related antibodies. The disease progression is chronic and closely resembles human Hashimoto’s thyroiditis. Further details about the phenotypic differences between NOD/LtJ and NOD.H-2^h4^ mice are summarized in **Table 1**.

**Table 1:** Differences between NOD/LtJ and NOD.H-2^h4^ mice.

Characteristic	NOD/LtJ mice	NOD.H-2^h4^ mice
Genetic background	Classical NOD strain (H-2^g7^)	NOD background + H-2^h4^ haplotype
Onset characteristics	experimental autoimmune thyroiditis (EAT)	spontaneous autoimmune thyroiditis (SAT)
Induction methods	Pig/human/mouse Tg+ Immune adjuvant	Feeding with high iodine water
Modeling time	6–8 weeks	8 weeks
Pathological manifestations	Thyroid follicular destruction and lymphocyte infiltration	Thyroid follicular destruction, extensive lymphocyte infiltration, germinal center formation, and interstitial fibrosis
Accessibility	Widely available	Jackson Laboratory

However, there are still several challenges in constructing animal models of AIT. For instance, there is variability in the dosage of thyroid antigens and immunization schedules across different laboratories (24, 25), and a standardized protocol has yet to be established. This lack of uniformity may lead to heterogeneity in disease models, making cross-study comparisons and result replication difficult. Researchers still need to invest significant time in exploring suitable modeling conditions. Secondly, the assessment of pathological processes, particularly in iodine-induced models, remains insufficient. There is a lack of long-term tracking studies on thyroid pathological changes following iodine induction, which hinders accurate prediction of disease progression. This is particularly important for studying long-term outcomes and evaluating therapeutic efficacy. Thirdly, the specificity of immune responses is another issue. In some models, the immune response may lack specificity, resulting in either insufficient or excessive reactions to thyroid antigens. This may not fully replicate the immunopathological features observed in humans.

To address these challenges, in this study, we designed experiments with varying injection methods, doses of thyroid antigens, adjuvants, and immunization schedules. Our aim is to comprehensively explore the impact of different immunization protocols on the construction of EAT mouse models. We will map the thyroid pathological landscape and changes in immune-inflammatory factors following multiple immunizations, revealing the dynamic regulatory mechanisms of disease progression under different immunization protocols. This will help researchers select the optimal modeling protocol in a shorter time, significantly improving experimental efficiency and reducing research costs. Furthermore, this study will lay the foundation for developing targeted immunotherapies, holding significant scientific and clinical value.

## 2 Materials and methods

### 2.1 Experimental animal

80 specific pathogen-free (SPF) female NOD/LtJ mice (6–8 weeks old; 20–25 g) were obtained from Beijing HFK Bioscience Co., Ltd. (Beijing, China) (License number: SCXK (Beijing) 2020-0004). The mice were housed in the Experimental Animal Center of Guang’anmen Hospital, China Academy of Chinese Medical Sciences, under standard conditions. They were provided with free access to food and water and maintained on a 12-hour light/dark cycle, with room temperature controlled at 20–22°C and relative humidity at 50%–60%. All procedures were conducted in accordance with the NIH Guide for the Care and Use of Laboratory Animals (2011) and the China Animal Management Regulations (Chinese Ministry of Health Document No. 55, 2001). The experimental protocol was approved by the Institutional Animal Care and Use Committee (IACUC) of Guang’anmen Hospital, China Academy of Chinese Medical Sciences (Ethical number: IACUC-GAMH-2022-031-01).

### 2.2 Animal grouping and immunization protocols

**Figure 1:**
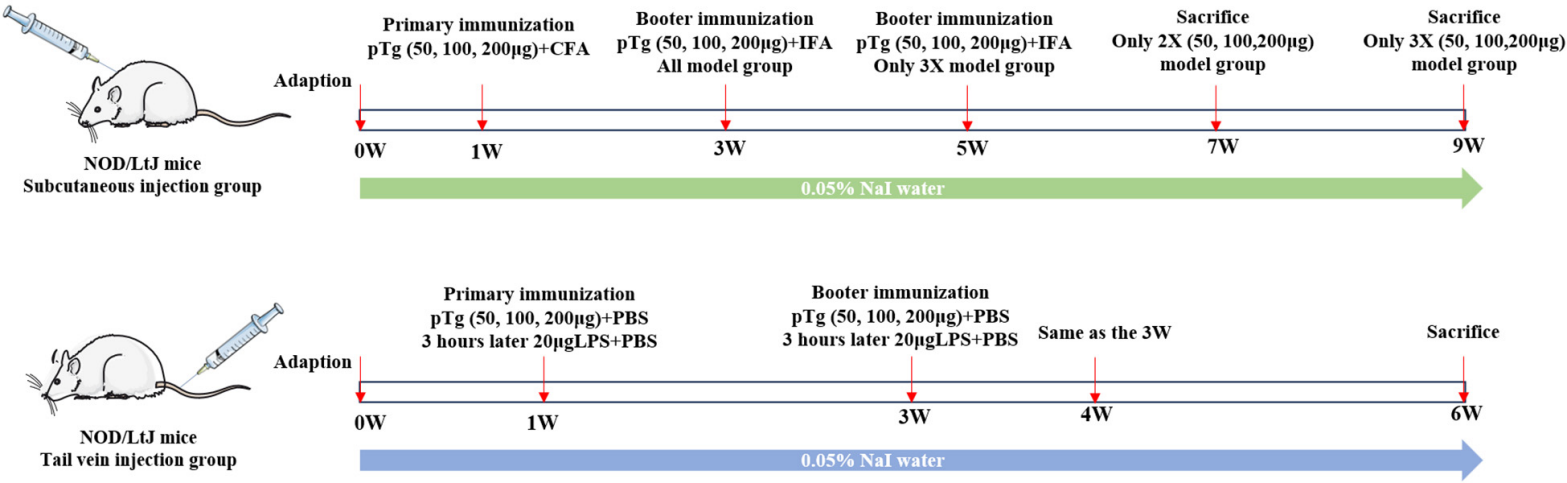
Comprehensive study flowchart.

A total of 80 female mice were randomly divided into two experimental modeling protocols: subcutaneous injection (n=48) and tail vein injection (n=32). Among these, the subcutaneous injection groups were randomly assigned to either control (C-2, C-3) or model groups (M-2X50, M-2X100, M-2X200, M-3X50, M-3X100, M-3X200), with 6 mice in each group. After 7 days of acclimatization, the model group was given sterile drinking water containing 0.05% NaI until the end of the experiment. The initial immunization was performed by administering multiple subcutaneous injections of porcine thyroglobulin (pTg, T1126, Sigma-Aldrich, USA) emulsified in CFA (F5881, Sigma-Aldrich, USA) into the dorsal cervical region of each mouse. Two weeks later, a booster immunization was administered using pTg emulsified in incomplete Freund’s adjuvant (IFA, F5506, Sigma-Aldrich, USA). Subsequently, a subset of mice received a third immunization at two-week intervals. The tail vein injection groups were randomly allocated to the control group (C-IV) and model groups (M-IV-50, M-IV-100, M-IV-200). After 7 days of acclimatization, the model groups were provided with sterile drinking water containing 0.05% NaI until the end of the experiment. Concurrently, each mouse in the model groups received an intravenous injection of pTg dissolved in phosphate-buffered saline (PBS, P1020, Solarbio, China), followed by an intravenous injection of LPS (L2630, Sigma-Aldrich, USA) dissolved in PBS 3 hours later. Using the same protocol, booster immunizations were administered at 2 weeks and 3 weeks after the initial immunization. Tissue samples were collected 2 weeks after the final injection. The experimental flowchart is shown in **Figure 1**, and the detailed experimental grouping and immunization protocols are shown in **Supplementary Table S1**.

### 2.3 Histopathological examination and evaluation of AIT mouse model

Hematoxylin-Eosin (HE) staining was performed to assess pathological changes in the thyroid tissues of mice. Fresh thyroid tissues were fixed in 4% paraformaldehyde for over 24 hours, followed by dehydration, paraffin embedding, and sectioning at 5 μm thickness. The degree of inflammatory infiltration in the thyroid tissues was observed under an optical microscope (Nikon, Japan). The mouse model was considered successfully established when the ratio of the area of lymphocyte infiltration to the total thyroid area exceeded 2%. The degree of lymphocytic infiltration in the thyroid was assessed and scored based on previously published studies (26): 0, no infiltration; 1, less than 10% lymphocytic infiltration of the thyroid; 2, 10-30% lymphocytic infiltration; 3, 30-50% lymphocytic infiltration; 4, greater than 50% lymphocytic infiltration. The final score was determined based on at least three non-consecutive thyroid sections.

### 2.4 Measurement of serum TPO-Ab and TG-Ab levels

The levels of TPO-Ab and TG-Ab in mouse serum were measured using enzyme-linked immunosorbent assay (ELISA). In line with previously published studies of similar design (27, 28), serum samples from six mice per group were detected. The serum was diluted fivefold, and the experimental procedures were conducted in accordance with the instructions provided by the ELISA kit (MEIMIAN, China).

### 2.5 Measurement of immune-inflammatory factors in mice

Serum levels of 12 mouse cytokines (IL-5, IL-22, IL-9, IL-10, IL-23p19, IL-13, IL-17A, IL-2, IL-6, IFN-γ, IL-4, TNF-α) were quantified using the RayPlex Mouse T Helper Cell Cytokine Array 1 Kit (FAM-TH-1, RayBiotech, USA) following the manufacturer’s protocol. Briefly, serum samples were diluted 4-fold with PBS and incubated with fluorescence-encoded beads coated with target-specific capture antibodies. After washing, biotinylated detection antibodies and PE-conjugated streptavidin were added to form a detection complex. Unbound reagents were removed via vacuum filtration using a 96-well filter plate. Beads were analyzed on a flow cytometer (equipped with blue and red lasers for APC and PE channels) to identify cytokine-specific bead populations and quantify PE median fluorescence intensity (MFI). Standard curves were generated using serial dilutions of lyophilized protein standards, and final cytokine concentrations were calculated via five-parameter logistic curve fitting. The raw absolute concentrations of cytokines were log-transformed and subsequently subjected to Z-score normalization in a row-wise manner. The processed data were then visualized using a clustered heatmap to elucidate the relationships within the dataset. The bioinformatics analysis was performed using the OECloud tools available at [https://cloud.oebiotech.com](https://cloud.oebiotech.com).

### 2.6 Multiplex immunofluorescence staining of thyroid tissues

Thyroid paraffin sections were dewaxed, rehydrated through graded ethanol series, and antigen-retrieved, followed by sequential Tyramide Signal Amplification (TSA) staining using primary antibodies against FOXP3 (1:5,000, Servicebio, GB115746), CD4 (1:5,000, Abcam, Ab183685), and ROR*γ*t (1:1,000, Abmart, TD3196S) incubated overnight at 4°C, corresponding HRP-conjugated secondary antibodies (Servicebio, GB23303) applied for 50 min at room temperature (RT), and TSA fluorophores incubated for 10 min at RT in the dark, with nuclei counterstained by DAPI (Servicebio, G1012); stained sections were imaged on a Nikon Eclipse C1 fluorescence microscope, where Th17 cells were quantified as CD4+/ROR*γ*t+ dual-positive cells (ROR*γ*t being the Th17-specific transcription factor) and Treg cells as CD4+/FOXP3+ dual-positive cells (FOXP3 being the Treg-specific transcription factor).

### 2.7 Immunohistochemistry of thyroid tissue

Formalin-fixed paraffin-embedded thyroid tissue sections (4 μm) were dewaxed in an eco-friendly dewaxing solution, rehydrated through graded ethanol, and subjected to antigen retrieval in citrate buffer. Endogenous peroxidase was blocked with 3% H_2_O_2_ (25 min, RT), followed by nonspecific site blocking with 3% BSA (30 min, RT). Sections were incubated overnight at 4°C with primary antibodies against IL-17A (1:300, Abcam ab79056), NLRP3 (1:300, Servicebio GB114320), and Caspase-1 (1:3,000, Servicebio GB15383), then probed with HRP-conjugated goat anti-rabbit IgG secondary antibody (Servicebio GB23303) for 50 min at RT. DAB chromogenic substrate (Servicebio G1212) was applied for signal development under microscopic monitoring, with nuclei counterstained by hematoxylin (Servicebio G1004). Sections were dehydrated in ethanol/n-butanol/xylene series and mounted with resin (Servicebio G1404). All images were acquired using a Nikon E100 bright-field microscope and quantitative analysis was performed using ImageJ software.

### 2.8 Statistical analysis

All data are presented as mean ± standard deviation (SD). Statistical analyses were performed using SPSS 25.0 (IBM, Armonk, NY, USA). Differences between groups were compared using one-way analysis of variance (ANOVA) followed by Tukey’s *post hoc* test at a 95% confidence level (P < 0.05). Graphical representations of the statistical results were generated using GraphPad Prism 9.

## 3 Results

### 3.1 Pathological inflammation and infiltration of mouse thyroid gland

**Figure 2:**
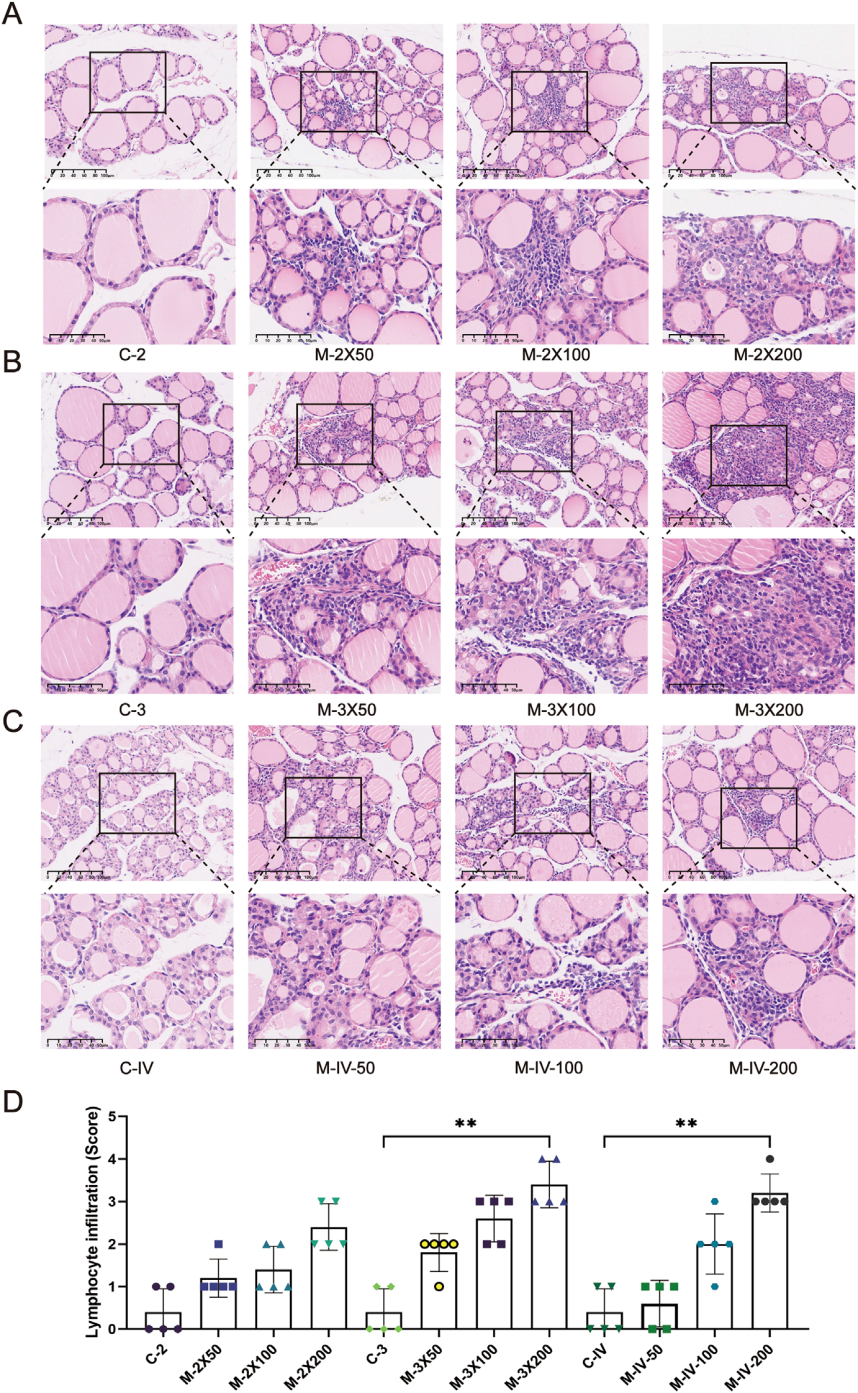
Histopathological changes in thyroid tissues of NOD/LtJ mice under different immune conditions. **(A)** HE staining shows thyroid inflammatory infiltration in mice after two subcutaneous injections. The normal control group (C-2) exhibits intact thyroid follicular structures with no significant lymphocyte infiltration. In the model groups (M-2X50, M-2X100, M-2X200), thyroid follicles are deformed or atrophied, with extensive lymphocyte infiltration around the follicles. Scale bar: 100 μm. **(B)** HE staining shows thyroid inflammatory infiltration in mice after three subcutaneous injections. The normal control group (C-3) displays intact and uniformly sized thyroid follicles with no significant lymphocyte infiltration. In the model groups (M-3X50, M-3X100, M-3X200), thyroid follicles are deformed or necrotic, surrounded by extensive lymphocyte infiltration. Scale bar: 100 μm. **(C)** HE staining shows thyroid inflammatory infiltration in mice after tail vein injection. The normal group (C-IV) exhibits intact and well-arranged thyroid follicles with no lymphocyte infiltration. In the model groups (M-IV-50, M-IV-100, M-IV-200), follicular structures are disrupted or atrophied, with necrotic thyroid epithelial cells and significant lymphocyte infiltration. Scale bar: 100 μm. **(D)** Statistical analysis of lymphocyte infiltration area scores for each group (n=5). Data are expressed as mean ± SD. ns: P > 0.05, *P < 0.05, **P < 0.01, ***P < 0.001.

Histopathological changes in the thyroid tissues of each group were evaluated using HE staining (**Figure 2**). In the control groups (C-2, C-3, C-IV), the thyroid follicles exhibited intact structures with uniform sizes, and no lymphocyte infiltration was observed (scores 0–1). In contrast, the model groups (M-2X50, M-2X100, M-2X200, M-3X50, M-3X100, M-3X200, M-IV-50, M-IV-100, M-IV-200) showed disrupted thyroid follicular structures and necrosis of thyroid epithelial cells. In severe cases, thyroid follicles were atrophied or completely absent (e.g., M-2X200, M-3X100, M-3X200). Except for the M-IV-50 group, lymphocyte infiltration was observed around the follicles in all other model groups, with infiltration areas ranging from 10% to over 50%, indicating that these model groups met the criteria for AIT disease models. The infiltration area scores increased with higher antigen doses, with the M-3X200 and M-IV-200 groups exhibiting the most severe lymphocyte infiltration (scores 3.4 ± 0.55 and 3.2 ± 0.45, respectively). These scores were significantly higher than those of the control groups (*p* < 0.01), suggesting that different antigen doses can influence the severity of inflammation, with a dose-dependent trend.

Furthermore, the degree of lymphocyte infiltration in the groups receiving two subcutaneous injections (M-2X50, M-2X100, M-2X200) was significantly lower than that in the groups receiving three subcutaneous injections at the same doses (M-3X50, M-3X100, M-3X200). This indicates that the frequency of immunization significantly affects the severity of inflammation. Under the same antigen dose, higher-frequency subcutaneous injections may lead to more pronounced thyroid inflammation. Additionally, at the same antigen dose (200 µg) and injection frequency, the inflammation score in the tail vein injection group (scores 3.2 ± 0.45) was slightly lower than that in the three subcutaneous injection group (scores 3.4 ± 0.55), suggesting that the injection method also influences thyroid inflammatory responses.

### 3.2 The levels of TPO-Ab and TG-Ab in mouse serum

**Figure 3:**
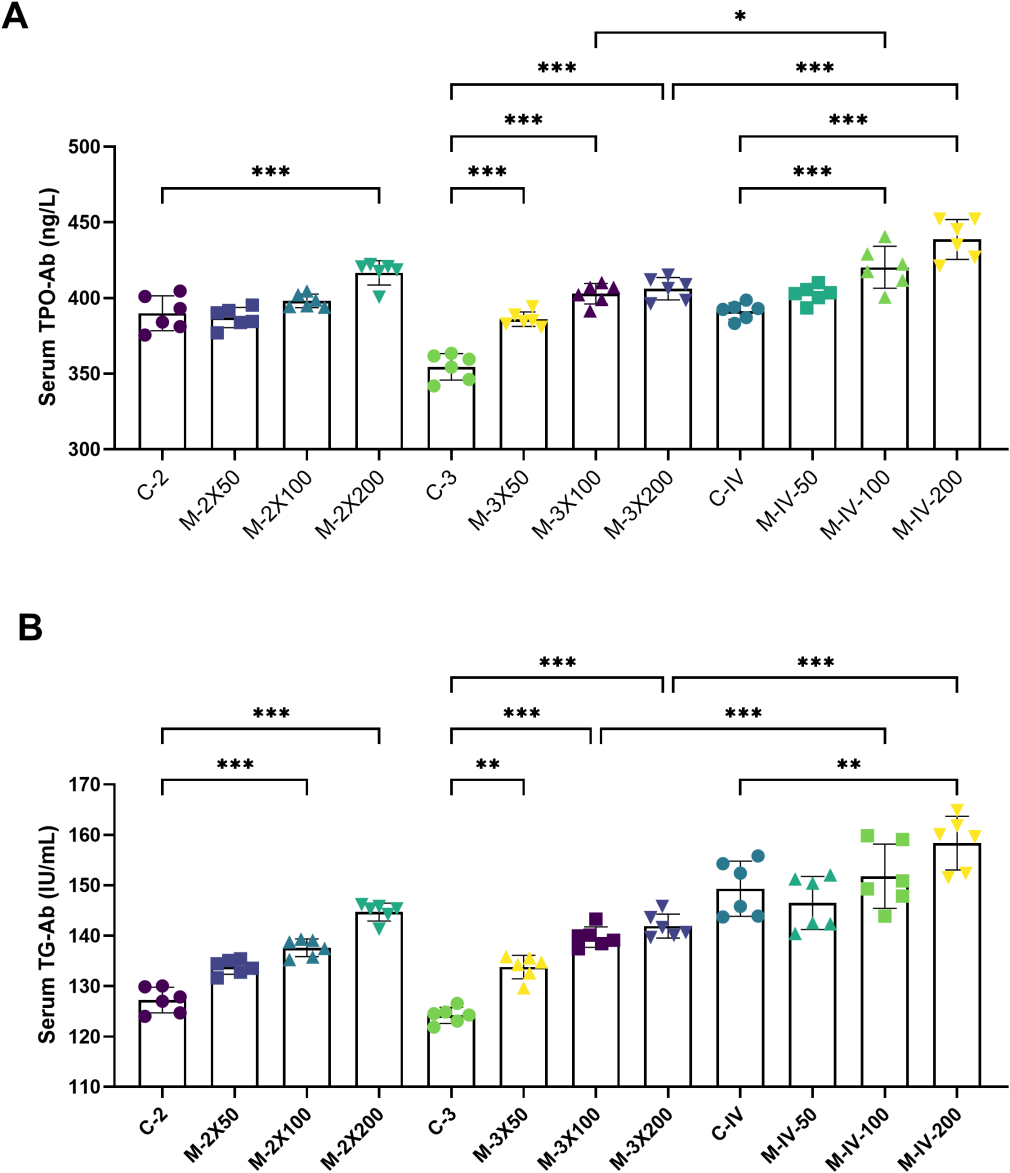
Serum TPO-Ab and TG-Ab levels in NOD/LtJ mice under different immunization conditions. **(A)** Compared to the normal groups (C-2, C-3, C-IV), the serum TPO-Ab levels in the model groups (M-2X50, M-2X100, M-2X200, M-3X50, M-3X100, M-3X200, M-IV-50, M-IV-100, M-IV-200) were significantly increased and showed a dose-dependent increase with higher antigen doses (n=6). **(B)** Compared to the normal groups (C-2, C-3, C-IV), the serum TG-Ab levels in the model groups (M-2X50, M-2X100, M-2X200, M-3X50, M-3X100, M-3X200, M-IV-50, M-IV-100, M-IV-200) were significantly elevated, with statistically significant differences, and were positively correlated with the injected antigen doses (n=6). Data are expressed as mean ± SD. ns: P > 0.05, *P < 0.05, **P < 0.01, ***P < 0.001.

ELISA results (**Figure 3A**) showed that the serum TPO-Ab level in the M-2X200 group was significantly higher than that in the C-2 group (*p* < 0.001). The serum TPO-Ab levels in the M-3X50, M-3X100, and M-3X200 groups were also significantly elevated compared to the C-3 group (*p* < 0.001), indicating that three immunizations had a more pronounced effect on serum TPO-Ab levels than two immunizations, and this effect was proportional to the antigen dose. In the tail vein injection groups, the TPO-Ab levels in both the M-IV-100 and M-IV-200 groups were significantly higher than those in the C-IV group (*p* < 0.001). The TPO-Ab level in the M-IV-200 group (438.8 ± 13.15) was notably higher than that in the M-3X200 group (406.2 ± 7.46) (*p* < 0.001), suggesting that the tail vein injection method induced a more sensitive serum thyroid antibody response compared to subcutaneous injection. Regarding serum TG-Ab levels (**Figure 3B**), Almost all subcutaneous injection model groups except for M-2X50 group showed significantly higher levels than their corresponding control groups (*p* < 0.001), with an increase in TG-Ab levels as the antigen dose increased. The TG-Ab level in the M-2X200 group (144.7 ± 1.77) was slightly higher than that in the M-3X200 group (141.9 ± 2.36) (*p* > 0.05), which may indicate that immunization frequency had no impact on TG-Ab changes. In the tail vein injection groups, only the M-IV-200 group exhibited a statistically significant difference in TG-Ab levels (158.4 ± 5.32) compared to the C-IV group (149.3 ± 5.47) (*p* < 0.01). Additionally, compared with the M-3X100 and M-3X200 groups, the M-IV-100 and M-IV-200 groups demonstrated significantly higher TG-Ab levels (all *p* < 0.001), further supporting the enhanced antibody production induced by tail vein injection.

### 3.3 Levels of immune inflammation related factors in mouse serum

**Figure 4:**
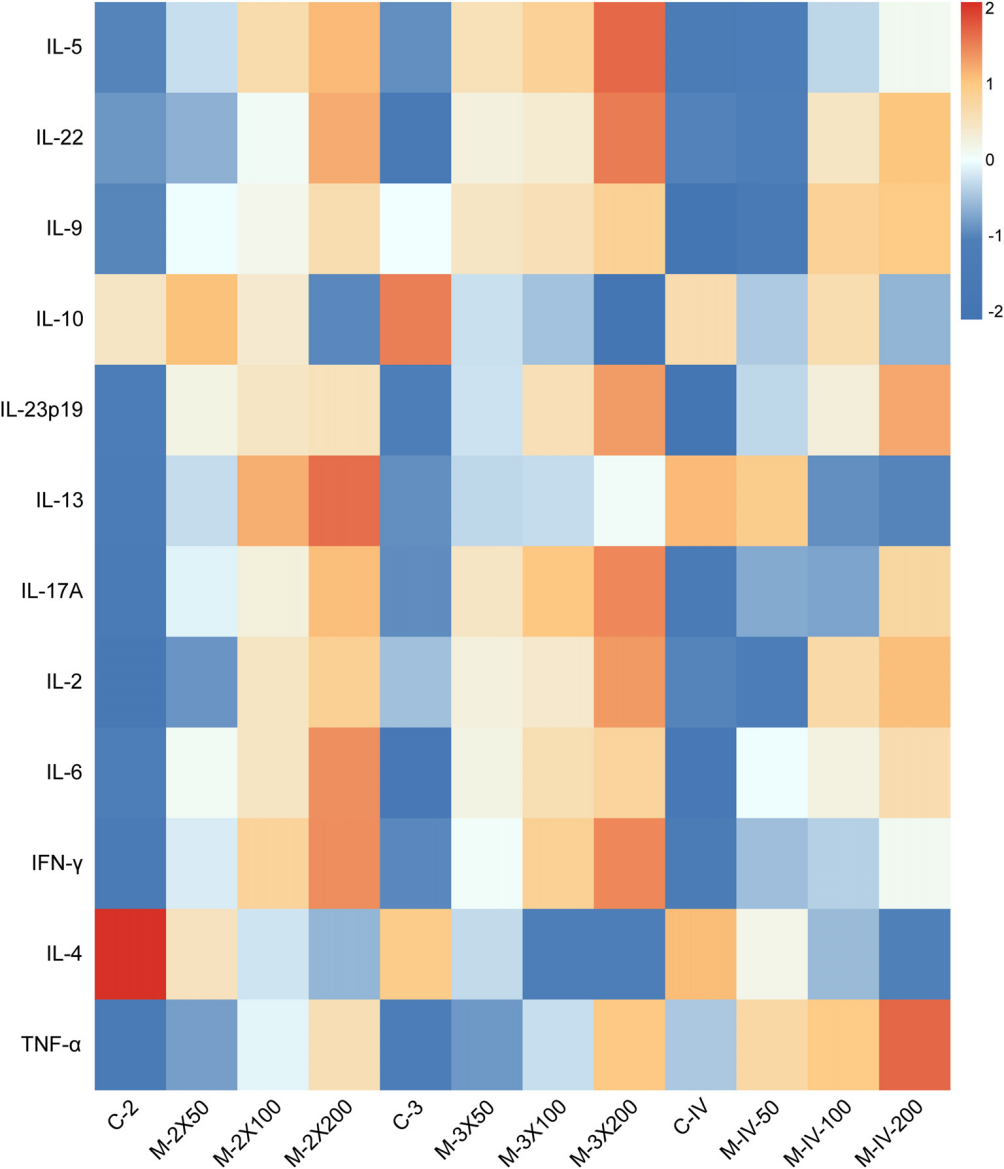
Heatmap of serum immune-inflammatory cytokine in NOD/LtJ mice under different immunization conditions. Data were preprocessed by log-transformation to stabilize variance, followed by row-wise Z-score normalization to enable cross-cytokine comparison. Color scale represents standard deviations from the mean expression of each cytokine. Absolute concentrations (pg/mL) are provided in **Supplementary Table S2**.

Using flow-based multiplex cytokine assay technology, the relative expression levels of 12 cytokines in mouse serum were detected (**Figure 4**). It was found that Th1/Th17-related cytokines (IL-17A, IL-6, TNF-α) were upregulated in the M-2X200, M-3X200, and M-IV-200 groups. In contrast, Th2-related cytokines (IL-10, IL-4) were downregulated in these groups. Additionally, the levels of IL-5, IL-22, IL-13, and IFN-γ in the subcutaneous injection groups were higher than those in the tail vein injection groups, while the TNF-α level was lower in the subcutaneous injection groups compared to the tail vein injection groups. These results suggest that different immunization routes may specifically activate distinct inflammatory pathways, leading to changes in related inflammatory factors.

### 3.4 Immune cell infiltration in thyroid tissue

**Figure 5:**
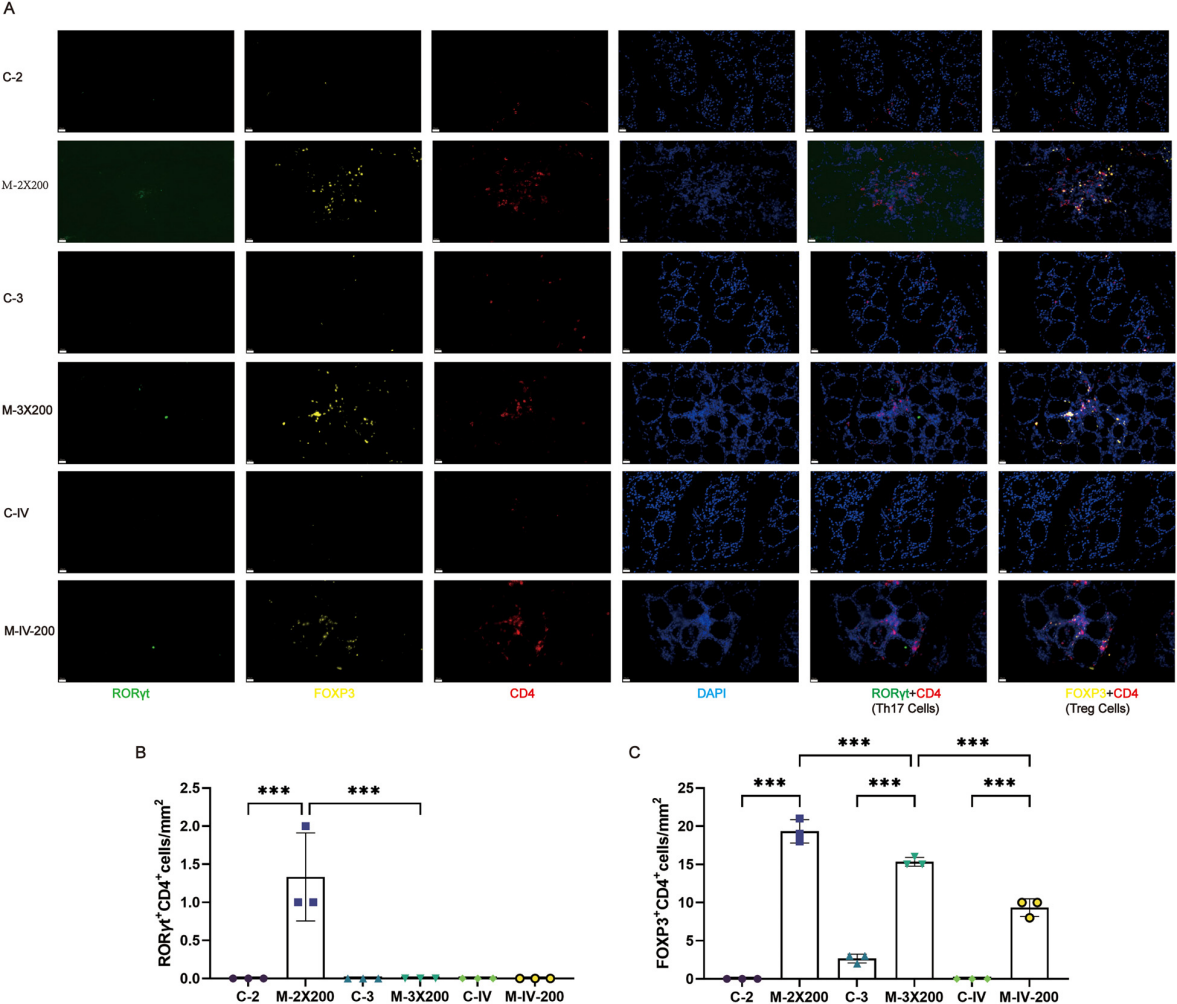
Multiplex immunofluorescence analysis of Th17 and Treg cell infiltration in thyroid tissues. **(A)** Immune cell infiltration in thyroid tissue under different immune conditions. Scale bar: 20 μm. **(B, C)** Multiplex immunofluorescence quantitative analysis (n=3). Data are expressed as mean ± SD. ns: P > 0.05, *P < 0.05, **P < 0.01, ***P < 0.001.

Based on pathological phenotypes and thyroid antibody expression profiles, the M-2X200, M-3X200, and M-IV-200 groups were selected to assess the impact of distinct immune conditions on local immune cell infiltration in thyroid tissues using multiplex immunofluorescence (**Figure 5A**). Cells co-expressing CD4 and ROR*γ*t were defined as Th17 cells (29), while those co-expressing CD4 and FOXP3 were defined as Treg cells (30). Th17 cell infiltration in thyroid tissues was minimal or absent across all immune conditions, with only trace infiltration observed exclusively in the M-2X200 group (**Figure 5B**). In contrast, Treg cells were significantly elevated in the M-2X200, M-3X200, and M-IV-200 groups compared to their respective controls (*p* < 0.001). However, increased immunization frequency reduced Treg cell density in the M-3X200 group relative to M-2X200 (*p* < 0.001). Furthermore, intravenous administration (M-IV-200) decreased Treg cells compared to the same-frequency/dose M-3X200 group (*p* < 0.001) (**Figure 5C**).

### 3.5 Cytokine activity in thyroid tissue

**Figure 6:**
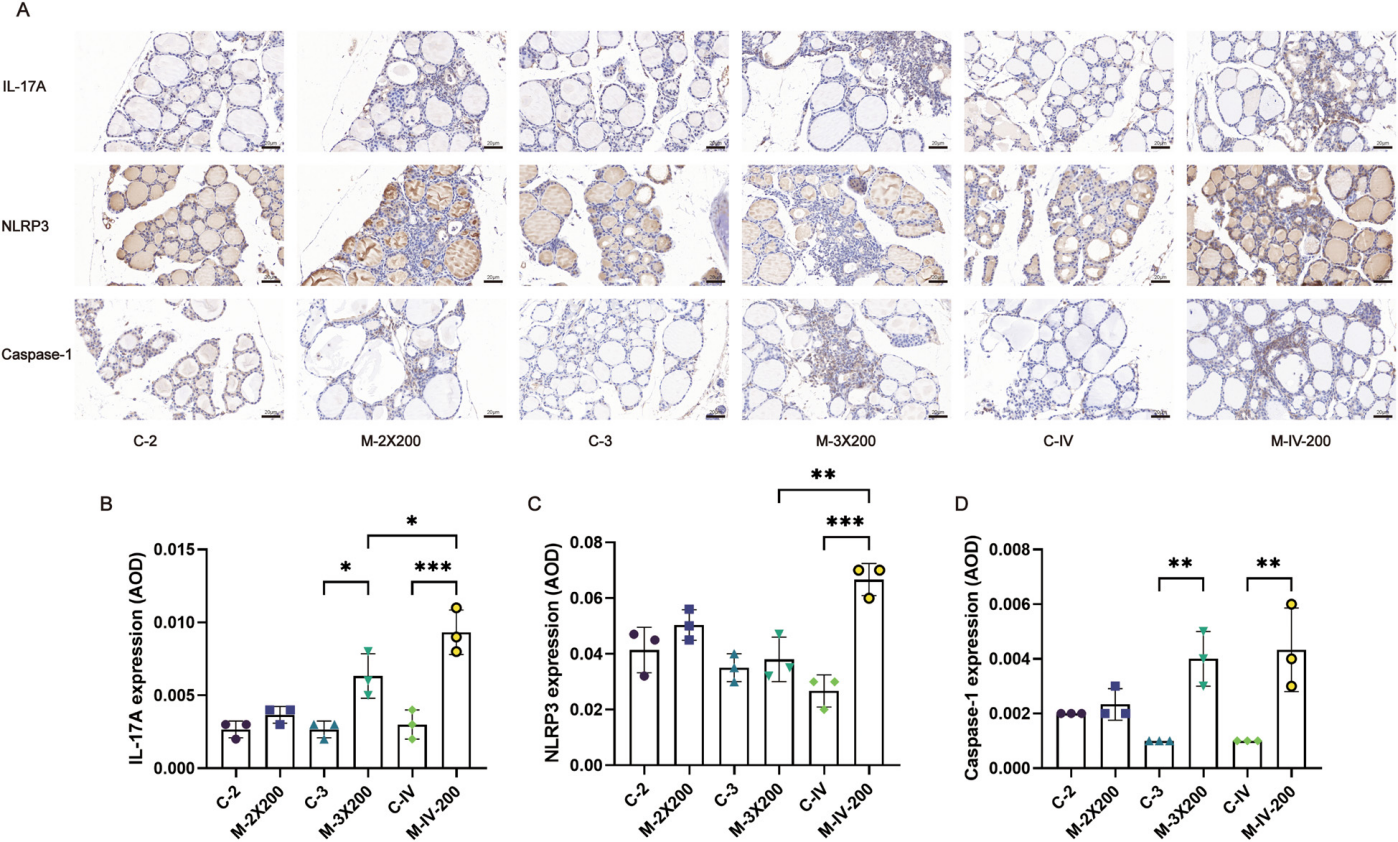
Immunohistochemical staining and quantitative analysis of IL-17A, NLRP3, and Caspase-1 in thyroid tissue. **(A)** Representative images of immunohistochemical staining for IL-17A, NLRP3, and Caspase-1 in mouse thyroid tissue. Scale bar: 20 μm. **(B–D)** Quantitative analysis of IL-17A, NLRP3, and Caspase-1 expression in thyroid tissue, bar graphs show the average optical density (AOD) of positive staining for each marker (n=3). Data are expressed as mean ± SD. ns: P > 0.05, *P < 0.05, **P < 0.01, ***P < 0.001.

Immunohistochemistry was employed to assess cytokine and inflammasome activity in murine thyroid tissues, elucidating the impact of distinct immune conditions on thyroid-specific immune signatures (**Figure 6A**). Results demonstrated significantly elevated IL-17A levels in the M-3X200 group compared to C-3 controls (*p* < 0.05), and markedly higher levels in M-IV-200 versus C-IV (*p* < 0.001). Moreover, M-IV-200 exhibited increased IL-17A expression relative to M-3X200 (*p* < 0.05) (**Figure 6B**). NLRP3 expression showed statistically significant upregulation exclusively in the M-IV-200 group (*p* < 0.001) (**Figure 6C**). Caspase-1 levels were significantly elevated in both M-3X200 and M-IV-200 groups compared to controls (*p* < 0.01) (**Figure 6D**).

## 4 Discussion

A successful animal model should closely resemble or simulate human disease pathology to facilitate research on disease mechanisms or pharmacology. In this study, the criteria for establishing an AIT model were based on internationally recognized standards, specifically a thyroid lymphocyte infiltration area-to-total gland area ratio of >2 (31). According to this criterion, all model groups except for the M-IV-50 group successfully established the AIT model. Additionally, based on clinical indicators observed in AIT patients, we measured the levels of serum thyroid antibodies in each group of mice and combined this with data on immune-related cytokines. After validation through multiplex immunofluorescence experiments and immunohistochemistry, we comprehensively assessed the pathological and immune changes in AIT animal models under different immune conditions (antigen dose, immunization frequency, and injection method). This approach aims to help researchers identify the optimal disease model from multiple perspectives. Our results demonstrated that high-dose antigen (200 μg pTg) combined with high-frequency (three immunizations) subcutaneous and intravenous injections significantly induced thyroid lymphocyte infiltration and elevated serum TPO Ab and TG Ab levels, showing the most pronounced changes compared to other groups. This method also promoted the activation of thyroid immune cytokines and inflammasomes. Furthermore, the tail vein injection method was more sensitive in promoting antibody production and activating specific inflammatory pathways (e.g.,NLRP3). By integrating existing literature with the findings of this study, we aim to provide an in-depth discussion on immune-inflammatory mechanisms, model optimization, and future research directions, offering valuable insights for researchers in this field.

### 4.1 Immunization frequency and modeling time

This study found that, under the same antigen dose, the thyroid inflammation infiltration scores and antibody levels in the three-immunization group were significantly higher than those in the two-immunization group. This may be related to the fact that multiple antigen injections can induce stronger immune memory, thereby enhancing antibody production and effector cell function (32). Additionally, repeated antigen stimulation can continuously activate antigen-presenting cells (APCs), leading to excessive immune system activation, promoting T/B cell activation, breaking immune tolerance, and ultimately resulting in increased autoantibody production and pro-inflammatory cytokine secretion (33, 34). However, high-frequency immunization may also induce systemic inflammatory responses, masking thyroid-specific pathological features (35). Therefore, under the premise of ensuring model stability, the three-subcutaneous-immunization protocol used in this study is more recommended. This approach not only effectively simulates the chronic thyroiditis disease process but also avoids excessive immune damage, achieving a balance between antigen stimulation and immune activation. Consistent with established protocols (24), thyroid tissues collected at 4 weeks post-immunization were subjected to pathological and immunological assessments. Prior evidence indicates that NOD-background mice lack self-remission capability and develop chronic lesions with aging, a phenomenon potentially linked to persistent Th1 responses and immune dysregulation (36). Nevertheless, multiple studies (37–39) have confirmed that significant lymphocyte infiltration and elevated serum antibodies meeting model establishment criteria are observable at this 4-week time point, aligning with our findings. Future investigations should incorporate longitudinal tracking of pathological changes beyond this acute phase, utilizing dynamic *in vivo* imaging or molecular markers at 8 or 12 weeks post-immunization to comprehensively evaluate disease progression and identify the optimal modeling time window.

### 4.2 Selection of injection methods

Subcutaneous injection and tail vein injection may differ in their immune response patterns. In this study, compared with the subcutaneous injection group, the intravenous tail injection group exhibited significantly higher levels of thyroid antibodies and more pronounced expression of IL-17A and NLRP3. This may be related to the direct activation of circulating immune cells (e.g., monocytes and dendritic cells) by intravenous LPS (40). Although the overall thyroiditis score in the intravenous tail injection group was slightly lower than that in the subcutaneous injection group, the local cellular immunity in the thyroid gland was similar to that observed with subcutaneous injection, both capable of stimulating the activation of T cell-related immune factors and causing an imbalance in Th17/Treg cell-mediated immunity. This indicates that when the antigen dosage reaches 200 μg, both local subcutaneous injection of thyroid antigens and intravenous injection can induce thyroid-targeted inflammation. It should be specifically noted that, due to limitations in experimental conditions, we were unable to investigate whether intravenous injection could achieve a similar level of targeted immune inflammation as subcutaneous injection when the thyroid antigen dosage is less than 200 μg, which represents a limitation of this study.

### 4.3 Antigen dose

In this study, as the antigen dose increased in both the subcutaneous and tail vein injection groups, the degree of inflammation and antibody levels gradually increased. Although there were no statistically significant differences in antibody levels between groups, the thyroid pathological manifestations in the 100μg and 200μg antigen groups showed obvious destruction or atrophy of thyroid follicular structures. This may be due to high-dose antigens promoting Th1/Th17 cell differentiation and the release of inflammatory factors such as IL-17A and NLRP3, leading to apoptosis of thyroid follicular epithelial cells and inflammatory infiltration, which directly causes follicular structure destruction (41). Additionally, studies have shown that CD8+ T cells can be activated under high-dose antigen stimulation, directly killing thyroid follicular cells through the perforin-granzyme pathway or the Fas/FasL pathway (42). Based on the degree of pathological inflammation, researchers can select an appropriate antigen dose according to their study objectives. Future research is still needed to further explore the pathological mechanisms of antigen-induced thyroid structural damage.

### 4.4 Immune mechanism

Our study found that the pathogenesis of thyroiditis induced by exogenous thyroglobulin injection in mice may be associated with T-cell imbalance and the activation of pro-inflammatory immune factors, which aligns with established research models. For instance, Ellis et al. (43) demonstrated that Treg dysfunction exacerbates thyroiditis, particularly highlighting that CD28 deficiency leads to reduced Treg numbers (characterized by low CD27/TNFR2/GITR expression), thereby impairing their ability to suppress autoreactive T cells and aggravating disease. Furthermore, Ippolito et al. (44) systematically compared the clinical, histological, and cytokine profile differences in thyroiditis induced by PD-1 versus CTLA-4 blockade. They observed that PD-1 inhibition triggered more severe thyroiditis with significantly elevated IL-6, whereas CTLA-4 blockade was associated with increased GM-CSF/MIP-1β and diffuse thyroid enlargement. This model not only validates the reproducibility of immune checkpoint inhibitor-associated thyroiditis but also provides a comparable paradigm of exogenous immune activation for our study. Additionally, Braley-Mullen et al. (45) demonstrated that B cells may act as antigen-presenting cells (APCs) to activate autoreactive T cells during early disease pathogenesis. In the absence of B cells at this critical stage, T cells remain inactivated or tolerant, even passive transfer of anti-MTg antibodies fails to induce thyroiditis in B-cell-deficient mice. This underscores the pivotal role of B cells in autoimmune thyroiditis. Future studies should incorporate B-cell surface marker analysis to elucidate the synergistic interplay between T and B cells in disease progression.

This study has several limitations: 1) Longitudinal tracking of immunization-induced pathological changes was not performed. Future investigations should incorporate dynamic imaging or molecular markers to comprehensively evaluate disease progression; 2) Gender-specific effects on the model remain uncharacterized. Subsequent experiments should include male cohorts to determine whether pathogenesis in NOD/LtJ mice exhibits sex-dependent characteristics; 3) Novel modeling approaches warrant exploration, including CRISPR-Cas9-engineered models with defined genetic backgrounds or stem cell-derived *in vitro* systems that better recapitulate human AIT pathology, thereby minimizing animal use while advancing humane research.

## 5 Conclusions

This study systematically compared the effects of different immunization conditions on the construction of the AIT model. By integrating pathological findings and thyroid antibody expression levels, all model groups, except for the M-IV-50 group, met the established criteria for model construction. Notably, subcutaneous and intravenous tail injection of 200 μg pTg administered three times induced more pronounced thyroid inflammatory infiltration and higher antibody levels in NOD/LtJ mice, while also demonstrating thyroid-specific immune activation. In summary, we recommend the immunization conditions of subcutaneous and intravenous tail injection of 200 μg pTg three times to better simulate the pathological process of AIT.

## Data Availability

The raw data supporting the conclusions of this article will be made available by the authors, without undue reservation.
