# Supplementary material for: Constructing the optimal experimental autoimmune thyroiditis mouse model using porcine thyroglobulin
Source: Front Immunol. 2025 Aug 20;16:1591196. doi: 10.3389/fimmu.2025.1591196 (PMC12405367; doi:10.3389/fimmu.2025.1591196)
Supplement: Supplementary file 1 [file Table1.docx]

Supplementary Material

# Supplementary Table S1

Grouping of experimental mice and detailed immunization protocols.

| Grouping | Immunization protocols |
| --- | --- |
| C-2 | At week 0, all mice were provided with sterile drinking water and received a 0.1 mL subcutaneous injection of an equal-volume mixture of PBS and CFA (without pTg) at multiple sites in the cervicodorsal region. At week 2, the same volume of PBS and IFA mixture (without pTg) was administered subcutaneously using an identical injection protocol. All animals were sacrificed for tissue collection at week 6. |
| M-2X50 | At week 0, mice were provided with sterile drinking water containing 0.05% NaI (500 mg/L). For primary immunization, 100 μg pTg was dissolved in an equal-volume mixture of 100 μL PBS and 100 μL CFA, followed by thorough emulsification to prepare a 0.5 mg/mL emulsion. Each mouse received a 0.1 mL subcutaneous injection of the emulsion (containing 50 μg pTg) at multiple sites on the dorsal neck region. At week 2, a booster immunization was administered using the same dose of pTg emulsified with IFA (containing 50 μg pTg) via identical injection protocol. All animals were sacrificed for tissue collection at week 6. |
| M-2X100 | At week 0, mice were provided with sterile drinking water containing 0.05% NaI (500 mg/L). For primary immunization, 200 μg of pTg was dissolved in 100 μl PBS and emulsified with an equal volume of CFA (100 μl) to form an emulsion at a final concentration of 1 mg/ml. Each mouse received a 0.1 ml subcutaneous injection (containing 100 μg pTg) at multiple sites on the dorsal neck region. At week 2, a booster immunization was administered using the same dose of pTg emulsified with IFA (containing 100 μg pTg). All animals were sacrificed for tissue collection at week 6. |
| M-2X200 | At week 0, mice were administered sterile drinking water containing 0.05% NaI (500 mg/L). For primary immunization, 400 μg of pTg was dissolved in 100 μl PBS and thoroughly emulsified with an equal volume of CFA (100 μl) to prepare an emulsion with a final concentration of 2 mg/ml. Each mouse received a 0.1 ml subcutaneous injection (containing 200 μg pTg) at multiple sites on the dorsal neck region. At week 2, a booster immunization was performed using the same dose of pTg emulsified with IFA (containing 200 μg pTg). All animals were sacrificed for tissue collection at week 6. |
| C-3 | At Week 0, mice were provided with sterilized drinking water and administered multipoint subcutaneous injections on the dorsal neck region (0.1 mL of equal-volume mixture of PBS and CFA without pTg). Booster immunizations with the same dosage of PBS and IFA (pTg-free) were administered subcutaneously at Weeks 2 and 4. All animals were sacrificed for tissue collection at week 6. |
| M-3X50 | At Week 0, mice were provided with sterile drinking water containing 0.05% NaI (500 mg/L). For primary immunization, 100 μg pTg was dissolved in an equal-volume mixture of 100 μL PBS and 100 μL CFA, followed by thorough emulsification to prepare a 0.5 mg/mL antigen emulsion. Each mouse received multipoint subcutaneous injections of 0.1 mL emulsion (containing 50 μg pTg) on the dorsal neck region. Booster immunizations with equivalent doses of pTg emulsified in IFA (containing 50 μg pTg) were administered subcutaneously at Weeks 2 and 4. All animals were sacrificed for tissue collection at week 8. |
| M-3X100 | At Week 0, mice were provided with sterile drinking water containing 0.05% NaI (500 mg/L). For primary immunization, 200 μg pTg was dissolved in an equal-volume mixture of 100 μL PBS and 100 μL CFA, followed by thorough emulsification to yield a 1 mg/mL antigen emulsion. Each mouse received multipoint subcutaneous injections of 0.1 mL emulsion (containing 100 μg pTg) on the dorsal neck region. Booster immunizations with equivalent volumes of pTg emulsified in IFA (containing 100 μg pTg) were administered subcutaneously at Weeks 2 and 4. All animals were sacrificed for tissue collection at week 8. |
| M-3X200 | At Week 0, mice were provided with sterile drinking water containing 0.05% NaI (500 mg/L). For primary immunization, 400 μg pTg was dissolved in an equal-volume mixture of 100 μL PBS and 100 μL CFA, followed by rigorous emulsification to prepare a 2 mg/mL antigen emulsion. Each mouse received multipoint subcutaneous injections of 0.1 mL emulsion (containing 200 μg pTg) on the dorsal neck region. Booster immunizations with equivalent volumes of pTg emulsified in IFA (containing 200 μg pTg) were administered subcutaneously at Weeks 2 and 4. All animals were sacrificed for tissue collection at week 8. |
| C-IV | At Week 0, mice were provided with sterile drinking water. Each mouse received an intravenous (IV) injection of 0.1 mL PBS via the tail vein, followed by a second 0.1 mL PBS injection using identical methodology 3 hours post-injection. Identical procedures with equivalent volumes of PBS (0.1 mL per injection) were administered through tail vein injection at Weeks 2 and 3. All animals were sacrificed for tissue collection at week 5. |
| M-IV-50 | At Week 0, mice were provided with sterile drinking water containing 0.05% NaI (500 mg/L). For primary immunization, 50 μg pTg was dissolved in 100 μL PBS to prepare a 0.5 mg/mL solution. Each mouse received an IV injection of 0.1 mL pTg solution (containing 50 μg pTg) via the tail vein, followed by a subsequent IV injection of 20 μg LPS dissolved in 100 μL PBS 3 hours post-initial injection. Booster immunizations using identical antigen doses (50 μg pTg + 20 μg LPS) and injection protocols were administered at Weeks 2 and 3. All animals were sacrificed for tissue collection at week 5. |
| M-IV-100 | At Week 0, mice were provided with sterile drinking water containing 0.05% NaI (500 mg/L). For primary immunization, 100 μg pTg was dissolved in 100 μL PBS to prepare a 1 mg/mL solution. Each mouse received an IV injection of 0.1 mL pTg solution (containing 100 μg pTg) via the tail vein, followed by a subsequent IV injection of 20 μg LPS dissolved in 100 μL PBS 3 hours post-initial injection. Booster immunizations using identical antigen doses (100 μg pTg + 20 μg LPS) and injection protocols were administered at Weeks 2 and 3. All animals were sacrificed for tissue collection at week 5. |
| M-IV-200 | At Week 0, mice were provided with sterile drinking water containing 0.05% NaI (500 mg/L). For primary immunization, 200 μg pTg was dissolved in 100 μL PBS to prepare a 2 mg/mL solution. Each mouse received an IV injection of 0.1 mL pTg solution (containing 200 μg pTg) via the tail vein, followed by a subsequent IV injection of 20 μg LPS dissolved in 100 μL PBS 3 hours post-primary injection. Identical immunization protocols (200 μg pTg + 20 μg LPS per administration) were repeated at Weeks 2 and 3. All animals were sacrificed for tissue collection at week 5. |
