# Supplementary material for: Constructing the optimal experimental autoimmune thyroiditis mouse model using porcine thyroglobulin
Source: Front Immunol. 2025 Aug 20;16:1591196. doi: 10.3389/fimmu.2025.1591196 (PMC12405367; doi:10.3389/fimmu.2025.1591196)
Supplement: Supplementary file 2 [file Table2.docx]

Supplementary Material

# Supplementary Table S2

Original absolute concentration of cytokines in each group.

| pg/ml | **C-2** | **M-2X50** | **M-2X100** | **M-2X200** | **C-3** | **M-3X50** | **M-3X100** | **M-3X200** | **C-IV** | **M-IV-50** | **M-IV-100** | **M-IV-200** |
| --- | --- | --- | --- | --- | --- | --- | --- | --- | --- | --- | --- | --- |
| IL-5 | 21.62 | 32.64 | 47.50 | 72.01 | 19.09 | 48.64 | 59.03 | 84.15 | 16.61 | 24.50 | 26.35 | 37.50 |
|  | 24.08 | 38.30 | 47.50 | 66.85 | 23.58 | 48.91 | 57.76 | 77.79 | 22.28 | 19.10 | 30.34 | 39.09 |
|  | 21.62 | 35.94 | 54.08 | 67.66 | 25.66 | 36.61 | 49.99 | 81.75 | 15.42 | 13.20 | 30.33 | 40.97 |
|  | 25.42 | 26.98 | 51.85 | 53.53 | 26.26 | 47.39 | 52.57 | 78.30 | 17.39 | 24.80 | 30.95 | 40.33 |
|  | 20.95 | 24.97 | 47.39 | 54.29 | 21.82 | 51.75 | 52.72 | 78.84 | 20.95 | 21.10 | 33.64 | 33.13 |
|  | 19.91 |  |  | 57.50 | 22.13 | 51.83 |  | 83.69 | 17.17 | 18.40 | 29.69 | 39.19 |
|  |  |  |  |  |  |  |  |  | 21.39 | 18.20 | 29.19 |  |
|  |  |  |  |  |  |  |  |  |  | 22.50 | 36.35 |  |
| **Mean valua** | **22.27** | **31.77** | **49.67** | **61.97** | **23.09** | **47.52** | **54.41** | **80.75** | **18.74** | **20.23** | **30.86** | **38.37** |
| IL-22 | 15.00 | 24.35 | 22.73 | 44.53 | 15.17 | 26.22 | 30.16 | 44.53 | 15.57 | 19.20 | 33.65 | 38.58 |
|  | 22.40 | 22.09 | 29.80 | 41.97 | 17.87 | 26.59 | 32.78 | 54.11 | 14.35 | 14.33 | 33.86 | 38.13 |
|  | 21.99 | 17.58 | 30.92 | 40.69 | 18.22 | 31.76 | 36.85 | 43.01 | 22.09 | 15.64 | 29.16 | 45.00 |
|  | 20.80 | 25.64 | 24.75 | 44.35 | 11.12 | 27.58 | 29.51 | 45.64 | 22.37 | 19.30 | 31.76 | 39.51 |
|  | 18.22 | 16.29 | 29.80 | 41.76 | 12.35 | 31.45 | 27.51 | 52.06 | 20.16 | 20.11 | 35.00 | 38.21 |
|  | 19.47 |  |  | 38.87 | 18.51 | 34.29 | 27.68 | 44.35 | 17.73 | 21.60 | 26.93 | 35.92 |
|  |  |  |  |  |  |  |  |  | 16.93 | 19.60 | 35.57 |  |
|  |  |  |  |  |  |  |  |  |  | 15.20 | 31.73 |  |
| **Mean valua** | **19.65** | **21.19** | **27.60** | **42.03** | **15.54** | **29.65** | **30.75** | **47.28** | **18.46** | **18.12** | **32.21** | **39.23** |
| IL-9 | 39.21 | 80.90 | 95.14 | 130.49 | 89.11 | 127.57 | 134.57 | 141.85 | 17.10 | 29.30 | 124.98 | 174.42 |
|  | 40.48 | 81.06 | 94.67 | 103.94 | 77.19 | 114.43 | 128.85 | 146.83 | 18.29 | 28.10 | 172.19 | 187.90 |
|  | 42.27 | 84.57 | 91.29 | 144.19 | 72.96 | 120.94 | 125.81 | 177.56 | 15.90 | 29.13 | 120.82 | 176.56 |
|  | 38.04 | 79.31 | 91.22 | 111.09 | 84.55 | 118.00 | 121.90 | 160.89 | 17.50 | 27.60 | 107.76 | 164.79 |
|  | 38.25 | 84.67 | 98.80 | 156.49 | 89.39 | 112.31 | 122.58 | 169.03 | 13.71 | 24.40 | 183.86 | 160.01 |
|  | 43.88 |  |  | 150.11 | 84.55 | 117.56 | 136.21 | 147.87 | 20.95 | 27.30 | 141.56 | 159.03 |
|  |  |  |  |  |  |  |  |  | 23.74 | 21.20 | 198.47 |  |
|  |  |  |  |  |  |  |  |  |  | 24.50 | 193.53 |  |
| **Mean valua** | **40.35** | **82.10** | **94.23** | **132.72** | **82.96** | **118.47** | **128.32** | **157.34** | **18.17** | **26.44** | **155.39** | **170.45** |
| IL-10 | 29.65 | 33.01 | 31.89 | 22.66 | 34.67 | 22.43 | 21.01 | 16.08 | 24.29 | 26.20 | 35.23 | 24.86 |
|  | 25.11 | 31.89 | 31.89 | 25.38 | 37.40 | 36.61 | 29.09 | 16.35 | 25.99 | 23.65 | 27.32 | 23.01 |
|  | 25.71 | 31.33 | 26.47 | 25.66 | 38.27 | 18.25 | 20.74 | 16.78 | 28.53 | 19.60 | 26.84 | 22.37 |
|  | 31.84 | 31.71 | 26.89 | 22.17 | 37.44 | 23.56 | 19.37 | 16.77 | 22.93 | 20.00 | 27.97 | 21.89 |
|  | 28.54 | 33.72 | 22.38 | 16.84 | 32.29 | 19.37 | 20.53 | 16.05 | 25.14 | 22.40 | 24.67 | 21.43 |
|  | 29.65 |  |  | 11.89 | 34.12 | 25.66 | 27.56 | 15.42 | 45.93 | 28.00 | 30.21 | 21.05 |
|  |  |  |  |  |  |  |  |  | 33.84 | 25.50 | 19.99 |  |
|  |  |  |  |  |  |  |  |  |  | 21.80 | 43.20 |  |
| **Mean valua** | **28.42** | **32.33** | **27.91** | **20.77** | **35.70** | **24.31** | **23.05** | **16.24** | **29.52** | **23.39** | **29.43** | **22.43** |
| IL-23p19 | 10.10 | 18.88 | 19.95 | 17.96 | 10.75 | 17.67 | 24.83 | 25.61 | 9.76 | 13.10 | 19.97 | 25.67 |
|  | 11.81 | 18.81 | 19.25 | 20.75 | 9.58 | 16.30 | 21.43 | 29.57 | 9.07 | 15.70 | 21.46 | 27.10 |
|  | 10.81 | 18.99 | 23.01 | 20.31 | 13.58 | 15.62 | 19.27 | 28.83 | 9.45 | 16.30 | 19.30 | 27.07 |
|  | 11.10 | 16.11 | 19.13 | 20.49 | 13.72 | 15.22 | 19.03 | 20.88 | 10.17 | 17.53 | 25.52 | 20.49 |
|  | 13.83 | 19.76 | 18.69 | 22.62 | 14.61 | 14.13 | 19.19 | 20.45 | 9.27 | 19.30 | 11.02 | 29.07 |
|  | 15.53 |  |  | 20.75 | 14.13 | 18.69 | 20.16 | 28.56 | 10.57 | 19.20 | 17.31 | 21.28 |
|  |  |  |  |  |  |  |  |  | 10.03 | 13.02 | 18.81 |  |
|  |  |  |  |  |  |  |  |  |  | 13.04 | 19.07 |  |
| **Mean valua** | **12.20** | **18.51** | **20.00** | **20.48** | **12.73** | **16.27** | **20.65** | **25.65** | **9.76** | **15.90** | **19.06** | **25.11** |
| IL-13 | 13.31 | 13.43 | 17.06 | 17.52 | 13.62 | 14.73 | 14.69 | 17.54 | 18.62 | 15.01 | 13.81 | 13.32 |
|  | 12.02 | 14.89 | 17.82 | 18.96 | 13.77 | 14.14 | 12.64 | 15.54 | 18.27 | 16.30 | 13.66 | 13.56 |
|  | 12.89 | 14.14 | 16.26 | 18.24 | 13.64 | 13.47 | 14.86 | 13.93 | 14.02 | 15.30 | 13.89 | 12.97 |
|  | 13.85 | 15.54 | 15.16 | 18.52 | 13.77 | 14.06 | 13.06 | 14.65 | 14.19 | 15.10 | 13.27 | 13.60 |
|  | 11.98 | 13.68 | 18.40 | 18.31 | 11.69 | 14.27 | 14.86 | 13.36 | 19.28 | 18.40 | 13.27 | 13.31 |
|  | 12.85 |  |  | 15.20 | 13.60 | 15.03 | 16.05 | 14.52 | 15.45 | 17.30 | 13.14 | 12.56 |
|  |  |  |  |  |  |  |  |  | 17.77 | 18.50 | 13.31 |  |
|  |  |  |  |  |  |  |  |  |  | 15.40 | 12.48 |  |
| **Mean valua** | **12.82** | **14.34** | **16.94** | **17.79** | **13.35** | **14.28** | **14.36** | **14.92** | **16.80** | **16.41** | **13.35** | **13.22** |
| IL-17A | 8.45 | 12.93 | 17.34 | 30.69 | 8.00 | 21.06 | 31.81 | 33.34 | 8.70 | 15.80 | 9.01 | 24.62 |
|  | 9.49 | 17.90 | 19.75 | 30.49 | 9.80 | 21.71 | 30.19 | 36.87 | 7.90 | 8.70 | 15.37 | 29.57 |
|  | 7.90 | 17.90 | 20.93 | 34.62 | 10.62 | 22.93 | 30.99 | 32.93 | 6.78 | 7.50 | 8.45 | 27.34 |
|  | 9.75 | 18.45 | 21.25 | 30.69 | 11.81 | 23.97 | 29.35 | 39.57 | 10.81 | 14.60 | 12.35 | 22.37 |
|  | 6.78 | 14.62 | 21.81 | 30.13 | 10.90 | 22.93 | 24.23 | 40.71 | 9.01 | 13.60 | 11.81 | 21.25 |
|  | 7.01 |  |  | 27.90 | 11.75 | 20.69 | 31.25 | 40.62 | 7.44 | 12.50 | 15.67 | 29.57 |
|  |  |  |  |  |  |  |  |  | 7.34 | 10.60 | 10.13 |  |
|  |  |  |  |  |  |  |  |  |  | 12.60 | 10.13 |  |
| **Mean valua** | **8.23** | **16.36** | **20.22** | **30.75** | **10.48** | **22.22** | **29.64** | **37.34** | **8.28** | **11.99** | **11.62** | **25.79** |
| IL-2 | 30.61 | 29.61 | 46.81 | 46.81 | 39.54 | 50.45 | 39.54 | 52.43 | 33.07 | 28.60 | 45.91 | 48.04 |
|  | 22.57 | 28.06 | 42.60 | 48.04 | 29.61 | 43.03 | 42.33 | 60.75 | 34.03 | 28.90 | 47.03 | 52.82 |
|  | 26.37 | 34.50 | 42.17 | 54.75 | 33.07 | 39.99 | 37.74 | 54.03 | 28.58 | 27.70 | 51.92 | 57.65 |
|  | 29.54 | 34.11 | 46.81 | 48.85 | 34.03 | 39.54 | 42.58 | 51.25 | 29.54 | 29.60 | 46.83 | 51.64 |
|  | 25.95 | 34.97 | 47.29 | 52.58 | 25.41 | 39.99 | 61.48 | 57.41 | 27.54 | 28.80 | 45.15 | 53.55 |
|  | 25.41 |  |  | 48.20 | 50.06 | 43.88 | 43.03 | 60.43 | 31.74 | 29.70 | 41.74 | 51.64 |
|  |  |  |  |  |  |  |  |  | 33.55 | 31.10 | 52.10 |  |
|  |  |  |  |  |  |  |  |  |  | 30.30 | 50.85 |  |
| **Mean valua** | **26.74** | **32.25** | **45.14** | **49.87** | **35.28** | **42.81** | **44.45** | **56.05** | **31.15** | **29.34** | **47.69** | **52.56** |
| IL-6 | 2.45 | 8.65 | 10.91 | 18.51 | 1.51 | 8.74 | 12.04 | 12.92 | 2.00 | 8.60 | 9.60 | 11.75 |
|  | 2.97 | 6.02 | 11.70 | 18.25 | 2.83 | 9.23 | 13.61 | 14.87 | 2.42 | 6.40 | 9.16 | 12.34 |
|  | 2.99 | 9.45 | 10.71 | 21.38 | 2.38 | 9.86 | 10.99 | 12.19 | 2.35 | 7.90 | 9.12 | 11.47 |
|  | 3.51 | 9.29 | 11.42 | 20.75 | 2.79 | 9.33 | 10.17 | 12.09 | 2.24 | 8.50 | 9.29 | 16.44 |
|  | 3.45 | 9.83 | 11.29 | 21.56 | 2.20 | 9.79 | 13.13 | 15.13 | 2.12 | 6.30 | 9.83 | 11.15 |
|  | 6.38 |  |  | 21.42 | 1.70 | 9.56 | 13.13 | 15.15 | 2.75 | 6.40 | 10.07 | 11.65 |
|  |  |  |  |  |  |  |  |  | 2.35 | 10.00 | 10.29 |  |
|  |  |  |  |  |  |  |  |  |  | 10.12 | 10.26 |  |
| **Mean valua** | **3.63** | **8.65** | **11.21** | **20.31** | **2.23** | **9.42** | **12.18** | **13.72** | **2.32** | **8.03** | **9.70** | **12.47** |
| IFN-γ | 4.23 | 10.51 | 21.13 | 31.76 | 5.63 | 13.32 | 21.20 | 31.33 | 3.62 | 8.10 | 9.43 | 12.26 |
|  | 4.92 | 11.65 | 20.98 | 35.72 | 5.02 | 11.53 | 19.31 | 33.94 | 4.34 | 6.70 | 9.06 | 14.92 |
|  | 3.26 | 10.51 | 21.18 | 31.94 | 6.48 | 13.59 | 21.89 | 32.43 | 4.51 | 11.30 | 9.13 | 11.55 |
|  | 3.85 | 10.43 | 21.13 | 32.20 | 5.33 | 11.64 | 22.03 | 31.08 | 3.94 | 9.70 | 8.33 | 13.65 |
|  | 3.96 | 10.29 | 20.67 | 32.03 | 5.79 | 12.32 | 25.21 | 34.51 | 5.67 | 9.10 | 9.28 | 12.30 |
|  | 3.87 |  |  | 30.29 | 6.59 | 11.13 | 21.23 | 34.43 | 4.40 | 5.30 | 8.92 | 13.01 |
|  |  |  |  |  |  |  |  |  | 4.06 | 7.50 | 9.20 |  |
|  |  |  |  |  |  |  |  |  |  | 7.50 | 9.26 |  |
| **Mean valua** | **4.01** | **10.68** | **21.02** | **32.32** | **5.81** | **12.26** | **21.81** | **32.95** | **4.36** | **8.15** | **9.08** | **12.95** |
| IL-4 | 24.13 | 9.20 | 6.96 | 6.11 | 13.90 | 6.10 | 4.70 | 3.63 | 14.41 | 13.30 | 9.20 | 4.13 |
|  | 26.68 | 10.93 | 7.80 | 9.20 | 13.20 | 8.11 | 6.71 | 4.13 | 12.69 | 9.90 | 8.00 | 4.98 |
|  | 24.98 | 9.52 | 9.48 | 5.83 | 14.76 | 8.13 | 8.36 | 5.83 | 16.68 | 9.40 | 5.55 | 5.27 |
|  | 18.08 | 12.40 | 9.63 | 6.96 | 13.31 | 9.97 | 3.56 | 8.08 | 15.14 | 8.80 | 7.13 | 8.64 |
|  | 22.11 | 18.64 | 9.68 | 8.92 | 13.27 | 9.12 | 3.66 | 6.92 | 18.08 | 9.00 | 6.96 | 6.96 |
|  | 23.27 |  |  | 6.98 | 18.31 | 8.98 | 7.24 | 6.96 | 15.55 | 10.40 | 6.40 | 5.83 |
|  |  |  |  |  |  |  |  |  | 16.40 | 10.50 | 6.84 |  |
|  |  |  |  |  |  |  |  |  |  | 11.20 | 9.20 |  |
| **Mean valua** | **23.21** | **12.14** | **8.71** | **7.33** | **14.46** | **8.40** | **5.70** | **5.92** | **15.56** | **10.31** | **7.41** | **5.97** |
| TNF-α | 125.15 | 145.05 | 193.09 | 240.79 | 132.50 | 147.38 | 161.02 | 281.62 | 177.22 | 204.53 | 264.17 | 322.85 |
|  | 135.10 | 152.85 | 193.22 | 260.42 | 135.05 | 157.13 | 178.43 | 281.55 | 162.35 | 265.80 | 284.70 | 327.86 |
|  | 137.66 | 163.96 | 180.93 | 248.64 | 130.93 | 150.26 | 174.63 | 283.19 | 185.71 | 253.10 | 267.41 | 316.15 |
|  | 117.24 | 159.15 | 194.35 | 233.69 | 138.93 | 146.31 | 180.63 | 260.13 | 174.44 | 262.10 | 279.03 | 395.73 |
|  | 123.00 | 153.96 | 207.86 | 233.96 | 140.93 | 156.79 | 208.93 | 265.09 | 174.34 | 271.80 | 269.79 | 322.74 |
|  | 115.71 |  |  | 233.96 | 136.88 | 159.84 | 196.15 | 278.93 | 174.67 | 265.30 | 273.00 | 370.56 |
|  |  |  |  |  |  |  |  |  | 165.24 | 242.10 | 271.46 |  |
|  |  |  |  |  |  |  |  |  |  | 237.80 | 267.24 |  |
| **Mean valua** | **125.64** | **154.99** | **193.89** | **241.91** | **135.87** | **152.95** | **183.30** | **275.09** | **173.42** | **250.32** | **272.10** | **342.65** |
